# Protein Kinase A in cellular migration—Niche signaling of a ubiquitous kinase

**DOI:** 10.3389/fmolb.2022.953093

**Published:** 2022-07-22

**Authors:** Kathryn V. Svec, Alan K. Howe

**Affiliations:** ^1^ Department of Pharmacology, University of Vermont, Burlington, VT, United States; ^2^ Department of Molecular Physiology and Biophysics, University of Vermont, Burlington, V T, United States; ^3^ University of Vermont Cancer Center, University of Vermont, Burlington, VT, United States

**Keywords:** protein kinase A, cell migration, subcellular signaling, compartmentalization, leading edge, Rho GTPases, ion channels, tyrosine kinases

## Abstract

Cell migration requires establishment and maintenance of directional polarity, which in turn requires spatial heterogeneity in the regulation of protrusion, retraction, and adhesion. Thus, the signaling proteins that regulate these various structural processes must also be distinctly regulated in subcellular space. Protein Kinase A (PKA) is a ubiquitous serine/threonine kinase involved in innumerable cellular processes. In the context of cell migration, it has a paradoxical role in that global inhibition or activation of PKA inhibits migration. It follows, then, that the subcellular regulation of PKA is key to bringing its proper permissive and restrictive functions to the correct parts of the cell. Proper subcellular regulation of PKA controls not only when and where it is active but also specifies the targets for that activity, allowing the cell to use a single, promiscuous kinase to exert distinct functions within different subcellular niches to facilitate cell movement. In this way, understanding PKA signaling in migration is a study in context and in the elegant coordination of distinct functions of a single protein in a complex cellular process.

## 1 Introduction

Cellular migration is an important process in many normal and pathophysiological biological functions from development to cancer metastasis. Migrating cells are constantly attuned to chemical and mechanical cues from the extracellular environment which regulate the mode, path, and extent of migration (Carter, 1965; Petrie et al., 2009). To efficiently move through the extracellular matrix (ECM), cells must sense and integrate these cues to iteratively build new attachments, sever old attachments, and push organelles and the cell body forward, all while constantly remodeling the cytoskeleton in a manner and direction that maximizes directionality (Ridley et al., 2003). Thus, cell migration is a balance of construction and deconstruction, protrusion and retraction, pushing and pulling, where polarity and the proper location of each of these actions is crucial for efficient movement. This intricate process requires the precise spatial and temporal coordination of myriad proteins and signaling pathways, working in concert to control cell shape and attachment (Ridley et al., 2003). Therefore, the signals and proteins that coordinate these functions must be present and active in specific, niche locations while absent or quiescent in others, and this distribution must be able to dynamically rearrange and adapt to changes in the chemical and mechanical microenvironment (Petrie et al., 2009). From the perspective of a single protein involved in cell migration, signaling is highly contextual—proper function is dependent on specific combinations of activators, inhibitors, partners, and substrates in precise locations within the cell, all of which may change as cell shape and position changes.

An example of such a contextual protein in cell migration is Protein Kinase A (PKA), a promiscuous serine/threonine kinase involved in innumerable cellular and biochemical processes. PKA is a heterotetrameric holoenzyme in which two catalytic subunits from two major families, Cα and Cβ (plus a third, rarer Cγ isoform) combine with homodimers formed by any of four R-subunits (RIα, RIβ, RIIα, RIIβ) to form a number of distinct, functionally nonredundant R2:C2 holoenzymes (Taylor et al., 2004; Taylor et al., 2005; Taylor et al., 2013; Taylor et al., 2022). Classically, however, two main subtypes of PKA are specified by the inclusion of either RI or RII regulatory subunits, each having nearly ubiquitous expression, but distinct allosteric properties, anchoring, and cellular localization, as expertly and extensively reviewed elsewhere (Taylor et al., 2013; Turnham and Scott, 2016; Gold, 2019; Michel and Scott, 2022; Taylor et al., 2022). Canonically, PKA is activated when cAMP binds to the regulatory subunits triggering release of the catalytic subunits [though recent work has challenged this cAMP gated free-release dogma (Smith et al., 2013; Smith et al., 2017; Isensee et al., 2018)] as reviewed in (Gold, 2019).

The importance of PKA activity and regulation for motile cellular behaviors has been demonstrated in myriad cell types: epithelia (e.g., Spurzem et al., 2002; Tkachenko et al., 2011); fibroblasts (e.g., Edin et al., 2001; Howe et al., 2005); endothelia (e.g., Kim et al., 2000; Nedvetsky et al., 2016; Adame-García et al., 2019); smooth muscle cells (e.g., Raymond et al., 2009; Hirakawa et al., 2007; Bornfeldt and Krebs, 1999); various leukocytes (e.g., Lang et al., 1996; Jones and Sharief, 2005; Canalli et al., 2007; Watson et al., 2015; Wehbi and Tasken, 2016; Jung et al., 2019); microglia and neurons (e.g., Kao et al., 2002; Golub and Caroni, 2005; Nasu-Tada et al., 2005; Han et al., 2007; Lee and Chung, 2009; Toriyama et al., 2012; Deming et al., 2015); and a wide variety of tumor cell lineages (e.g., (O'Connor and Mercurio, 2001; Paulucci-Holthauzen et al., 2009; McKenzie et al., 2011; Shaikh et al., 2012; Armaiz-Pena et al., 2013; Burdyga et al., 2013; Ko et al., 2013; Feng et al., 2014; Ou et al., 2014; Feng et al., 2015; Barquilla et al., 2016; Hung et al., 2016; Bensalma et al., 2019; Jung et al., 2019; Tonucci et al., 2019; Huang et al., 2020; Jiang et al., 2020; McKenzie et al., 2020; Han et al., 2021). As in many of its other functional milieus, PKA has both a positive and negative role in cellular migration, depending on the context (Diviani and Scott, 2001; Howe, 2004). Moreover, PKA and its substrates can be found in virtually every dark corner of a cell, and the list of known PKA substrates numbers in the high hundreds with new additions added regularly (Shabb, 2001; Ruppelt et al., 2009). Therefore, the activity of PKA and the location of that activity needs to be controlled tightly for cell migration to progress (Howe, 2004). This facet of PKA regulation is achieved through its association with A Kinase Anchoring Proteins (AKAPs) which serve to anchor PKA to specific locations within the cell (Diviani and Scott, 2001; Omar and Scott, 2020). Further, AKAPs scaffold higher order signaling complexes and juxtapose PKA, proteins involved in regulating PKA activity, and potential targets of PKA activity (Michel and Scott, 2002). To this end, cell migration requires not only regulation of PKA activity but also specific localization of that activity (Lim et al., 2008; Paulucci-Holthauzen et al., 2009; McKenzie et al., 2011). Biochemical and image-based experimentation has identified active PKA in the leading edge of migrating cells, putting it in the vicinity of many confirmed and suspected substrates in actin and adhesion dynamics and other spatially coordinated efforts in cell migration (Howe et al., 2005; Lim et al., 2008; Paulucci-Holthauzen et al., 2009; McKenzie et al., 2011; Tkachenko et al., 2011; McKenzie et al., 2020). Further, this leading edge PKA activity is sensitive to changes in actomyosin contractility (McKenzie et al., 2020). Thus, PKA is a prime example of a signaling node in migration that is highly contextual.

The goal of this review is not to summarize every known or putative target of PKA in migration or to present an exhaustive list of studies in this milieu. Other reviews have tackled these lofty topics more completely (Diviani and Scott, 2001; Howe, 2004). Rather, this review will draw attention to the need for spatial organization of PKA activity during the specialized cellular function of migration and some of the progress that has been made in this area. Principally, our focus stems from the question ‘How is localized signal transduction achieved during migration?’—Indeed, PKA may be used as a case study in this respect. Lessons learned regarding the highly contextual regulation of this pleiotropic protein kinase will shed light on how the cell is able to regulate other far-reaching enzymes during migration and other complex processes. Here, we will consider where PKA is found, what some of its major targets are, and how it is regulated in the context of cell migration.

## 2 Location, location, location

Several studies have shown the importance of PKA localization for cell migration, as reviewed in (Howe, 2004; 2011). With AKAPs pinning PKA to many diverse structures within the cell, it is clear that AKAPs have an important role in PKA’s localization in this context. Pertinent to this review, several AKAPs have been identified that associate with the actin cytoskeleton (Diviani and Scott, 2001) and cell membrane (Burgers et al., 2012), delivering PKA to locations involved in migration. Functionally, once PKA is localized, it is the location of PKA *activity* that dictates its role in migration. While other reviews have comprehensively discussed AKAPs associated with the cytoskeleton (Diviani and Scott, 2001), the focus of this section is on the detection of PKA activity in distinct subcellular compartments relevant to migration.

### 2.1 The leading edge

The leading edge is the foremost protrusive structure, leading the cell with a dense, growing actin meshwork (Abercrombie et al., 1970; Pollard and Borisy, 2003; Ridley et al., 2003; Ridley, 2011). The best characterized pool of PKA activity in migrating cells is that in the leading edge. An early biochemical study found PKA RII subunit and PKA activity are both present in higher amounts in protrusive pseudopodia than in the cell body during chemotaxis (Howe et al., 2005). This agrees with the localization of both PKA RI (Lim et al., 2007) and RII (Howe et al., 2005) subunits in the leading edge as visualized via immunofluorescence. Surprisingly, though PKA was more active in pseudopods, there was not more PKA catalytic subunit there than in the cell body (Howe et al., 2005), which points to local differential regulation of PKA in the leading edge.

**FIGURE 1:**
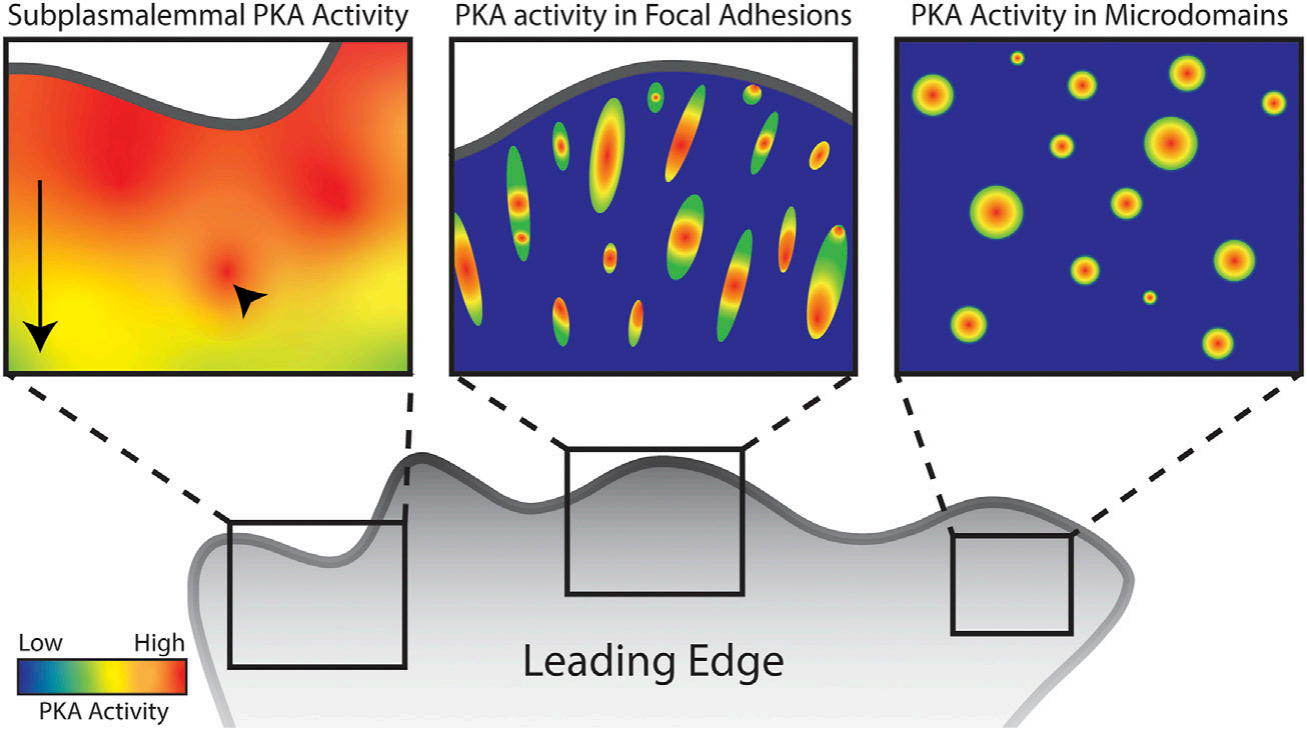
Schematic of PKA activity localized to distinct subcellular regions and structures in a migrating cell. Arrow highlights overall front to back gradient of subplasmalemmal PKA activity in the leading edge while arrowhead highlights hotspots of PKA activity. Though leading edge PKA activity has been well characterized, there are very few studies characterizing PKA activity in focal adhesions and microdomains. Images serve as representation of the concentration of PKA activity in these structures.

Since the advent of the AKAR series of FRET based biosensors specific for PKA activity (Zhang et al., 2001; Zhang et al., 2005; Allen and Zhang, 2006), many groups have characterized the dynamics of PKA in migrating cells. Confirming the above findings, AKAR biosensors have revealed strong PKA activity in the leading edge of several cell types (Lim et al., 2008; Paulucci-Holthauzen et al., 2009; McKenzie et al., 2011; Tkachenko et al., 2011; McKenzie et al., 2020). Notably, leading edge PKA activity is best detected by biosensors targeted to the plasma membrane, in both raft and non-raft domains (McKenzie et al., 2020). Further, at least one report shows that this activity is present only at the basal membrane in a two-dimensional imaging system (Paulucci-Holthauzen et al., 2009). This pool of PKA activity is often described as a gradient, with generally high PKA activity at the leading edge that diminishes toward the cell body. Upon closer examination, there are peaks in PKA activity within this gradient that are separable and dynamic (Figure 1, left panel). Morphodynamic studies revealed that peaks in leading edge PKA activity are spatially and temporally correlated with protrusion dynamics. This control of protrusion-retraction cycles by PKA occurs through the phosphorylation of RhoA, as discussed later (Tkachenko et al., 2011).

Still other studies have revealed that leading edge PKA activity is mediated by integrins, extracellular matrix receptors, as integrin-specific peptides can block the formation of PKA gradients and events in the leading edge (Lim et al., 2008). In fact, striped, patterned extracellular matrix (ECM) underlying adhesive cells leads to correspondingly striped appearance of leading edge PKA activity, exhibiting a strikingly similar pattern above sites of adhesion to the ECM (Tkachenko et al., 2011).

Though PKA holoenzymes can associate with the plasma membrane without AKAP function (Zhang et al., 2015), leading edge PKA activity is dependent on type II AKAP anchoring as shown by disruption of canonical AKAP anchoring to PKA RII using the Ht31 peptide leading to ablation of the leading edge PKA gradient and membrane protrusion (Paulucci-Holthauzen et al., 2009). This study further identifies that the AKAP-Lbc significantly contributes to leading edge PKA gradients but is presumably not the only AKAP involved as knockdown only partially disrupted leading edge PKA activity (Paulucci-Holthauzen et al., 2009).

Finally, leading edge PKA activity can be induced in relatively non-motile HeLa cells by simply asymmetrically recruiting RII regulatory subunit to the plasma membrane. This study used a rapamycin inducible recruitment system to recruit R subunit to the plasma membrane and found that at moderate levels of recruitment, PKA activity was increased, and the cell would move toward the rapamycin gradient (LaCroix et al., 2022). At high levels of R subunit recruitment, PKA activity was ultimately inhibited. Interestingly, in this case, gradients of PKA activity formed in the obverse direction and cells moved away from rapamycin stimulation. This suggests that simply changing the ratio of regulatory to catalytic subunit can alter PKA signaling and even induce leading edge PKA activity and cellular movement.

It’s important to note that, given the exclusive use of membrane-targeted AKAR biosensors, these studies describe PKA activity that is occurring solely at the leading edge plasma membrane. The identification and characterization pools of PKA activity in the bulk cytoplasm and other discrete locations during cell migration have yet to be as thoroughly explored.

### 2.2 Integrin based adhesive structures

Integrins are extracellular matrix receptors that span the plasma membrane and act as nucleators for focal adhesion structures (Legerstee and Houtsmuller, 2021). Focal adhesions (FAs) are rich protein complexes that anchor the actin cytoskeleton to integrins. Focal adhesions are packed with known and putative targets of phosphorylation by PKA, examples of which are discussed later. Given PKA’s established role in actin-based migration and activity near the membrane, it follows that PKA is likely located within focal adhesions (Figure 1, center panel).

α4β1 integrins have been identified as noncanonical AKAPs for Type I PKA (Lim et al., 2007). Specifically, the cytoplasmic tail of the α4 integrin anchors the entire PKA holoenzyme in a manner that is not disrupted by common AKAP disrupting peptides. Though the binding site was not specifically identified, the interaction between PKA and α4 did not disrupt and was not affected by binding of paxillin to α4, one of the primary interactions in the formation of focal adhesions (Lim et al., 2007). Thus, PKA is localized to at least some integrin-based adhesive structures via a noncanonical AKAP interaction with α4 integrins.

Though focal adhesion complexes are tightly associated with the plasma membrane, membrane-targeted PKA biosensors show that leading edge gradients and hotspots of PKA activity aren’t directly correlated with adhesion structures (McKenzie et al., 2020). However, in addition to α4 integrin-mediated anchoring, PKA regulatory and catalytic subunits have been reported in a variety of focal adhesion proteomes, prepared from a variety of cells using distinct methods (Zaidel-Bar et al., 2007; Kuo et al., 2011; Schiller et al., 2011; Horton et al., 2015), strongly suggesting a specific FA pool of PKA. Despite this, there are no reported studies observing PKA activity directly within focal adhesions themselves. Clearly, further work must be done to elucidate how PKA is anchored to focal adhesions and the targets and consequences of PKA activity within them during migration.

### 2.3 Other locations

Several other locations or structures pertinent to migration have been identified as local PKA hotspots. Namely actin-based protrusive structures and smaller micro domains (Figure 1, right panel).

Invadopodia and podosomes are specialized projections involved in the degradation of local extracellular matrix material, clearing the way for cell migration (Murphy and Courtneidge, 2011; Ridley, 2011). Active, phosphorylated PKA has been found in invadopodia and is upstream of proteolytic invadopodia activity (Debreova et al., 2019). Further, PKA activity promotes the formation of invadopodia (Tonucci et al., 2019). While one study in adrenal cells demonstrates a dependence of podosome formation on PKA activity (Colonna and Podestá, 2005), another study in angiogenic sprouting shows an antagonistic effect of PKA on podosome rosette formation (MacKeil et al., 2019).

Even smaller actin-based structures have also been found to contain PKA activity. Filopodia are fine, actin-based, probing protrusive structures in the leading edge. These structures are important for guiding cells and sensing mechanical inputs (Mattila and Lappalainen, 2008; Ridley, 2011; Bornschlögl, 2013; Heckman and Plummer, 2013; Jacquemet et al., 2015). Signaling through PKA is important for the formation of these structures (Gomez and Robles, 2004; Deming et al., 2015). Type II PKA localized to neuronal growth cone filopodia through AKAP binding encourages growth cone mobility and turning (Han et al., 2007). Further, tethering to AKAPs—for example Gravin (RII) (Burgers et al., 2012) and smAKAP (RI) (Nauert et al., 1997)—has been shown to be important for PKA localization and function in filopodia in different cell types. Similarly, microspikes, which are akin to filopodia but reside within the veil of the lamellipodium at the leading edge of migrating cells and on neuronal growth cones, display PKA RII subunit tightly associated with actin structures (Rivard et al., 2009). This association is independent of canonical AKAP function and follows the dynamic nature of the actin structures themselves. This study not only identifies PKA localized to protrusive actin, but points to a more direct coupling of the kinase to the actin cytoskeleton, without the use of AKAPs or the AKAP binding motif. Given the small size and highly specialized function of these cellular structures, it is likely that PKA activity has heretofore unexplored functions within filopodia and microspikes.

Going further down the scale of cellular structures, lipid rafts and membrane microdomains such as caveolae are historically and intimately associated with the regulation of migration (Golub and Caroni, 2005; Head et al., 2014). Intriguingly, such microdomains have been reported to contain many components of the canonical PKA signaling pathway–adenylyl cyclases, phosphodiesterases, and PKA itself–often scaffolded together by AKAPs or other adapters (Head et al., 2006; Swaney et al., 2006; Patel et al., 2008). However, despite the aforementioned presence of PKA signaling machinery in microdomains and some elegant observations of microdomain regulation by PKA (Golub and Caroni, 2005), the contribution of raft- or caveoli-associated PKA signaling to migration remains largely unexplored. At an even smaller scale, a recent study characterized droplets of liquid-liquid phase separated RII which concentrate cAMP/PKA signaling (Zhang et al., 2020). These microdomains contained elevated PKA activity and sequestration of cAMP. The effect of this sequestration of signaling and effector molecules is to concentrate PKA activity for local signaling and prevent dissociation of cAMP and degradation by phosphodiesterases (Zhang et al., 2020). This type of microdomain presents evidence of AKAP independent concentration of PKA signaling which could dynamically regulate local signaling during a kinetic and iterative process such as cell migration.

Finally, the smallest possible ‘location’ at which PKA signaling specificity and localization can occur is at the protein-level–specifically, the sphere of targets within molecular proximity of the enzyme itself. AKAPs serve as a nano scaffolds that bring together PKA, substrates, and regulatory proteins such as phosphodiesterases (Omar and Scott, 2020), supporting a discrete complex that is ‘hard-wired’ to focus and control the PKA activity within. It is at the level of anchoring that local PKA activity is truly specified and connected with its substrates. Further, recent evidence shows PKA may function and be regulated in a hyper-local manner. These studies reveal that PKA catalytic subunits are catalytically active without completely dissociating from the regulatory subunits. This finding highlights the importance and sophistication of the anchoring of PKA to precise locations and exquisitely increases spatial regulation of target specificity (Smith et al., 2013; Smith et al., 2017; Isensee et al., 2018). Given both the importance and the scales of localized PKA function, characterization of the specific targets regulated by these discrete pools of activity is of considerable importance.

## 3 Targets of PKA activity

PKA is delivered to different subsets of targets by nature of the high specificity of anchoring and localization of activity, as discussed in other sections. As a ubiquitous kinase, PKA has innumerable targets, many of which are associated with adhesion or migration. Given that PKA activity has been most heavily studied near the membrane, identifying membrane targeted substrates is where the most progress has been made. There are many intriguing examples of such targets, a few of which will be discussed here.

### 3.1 Rho GTPases

**FIGURE 2:**
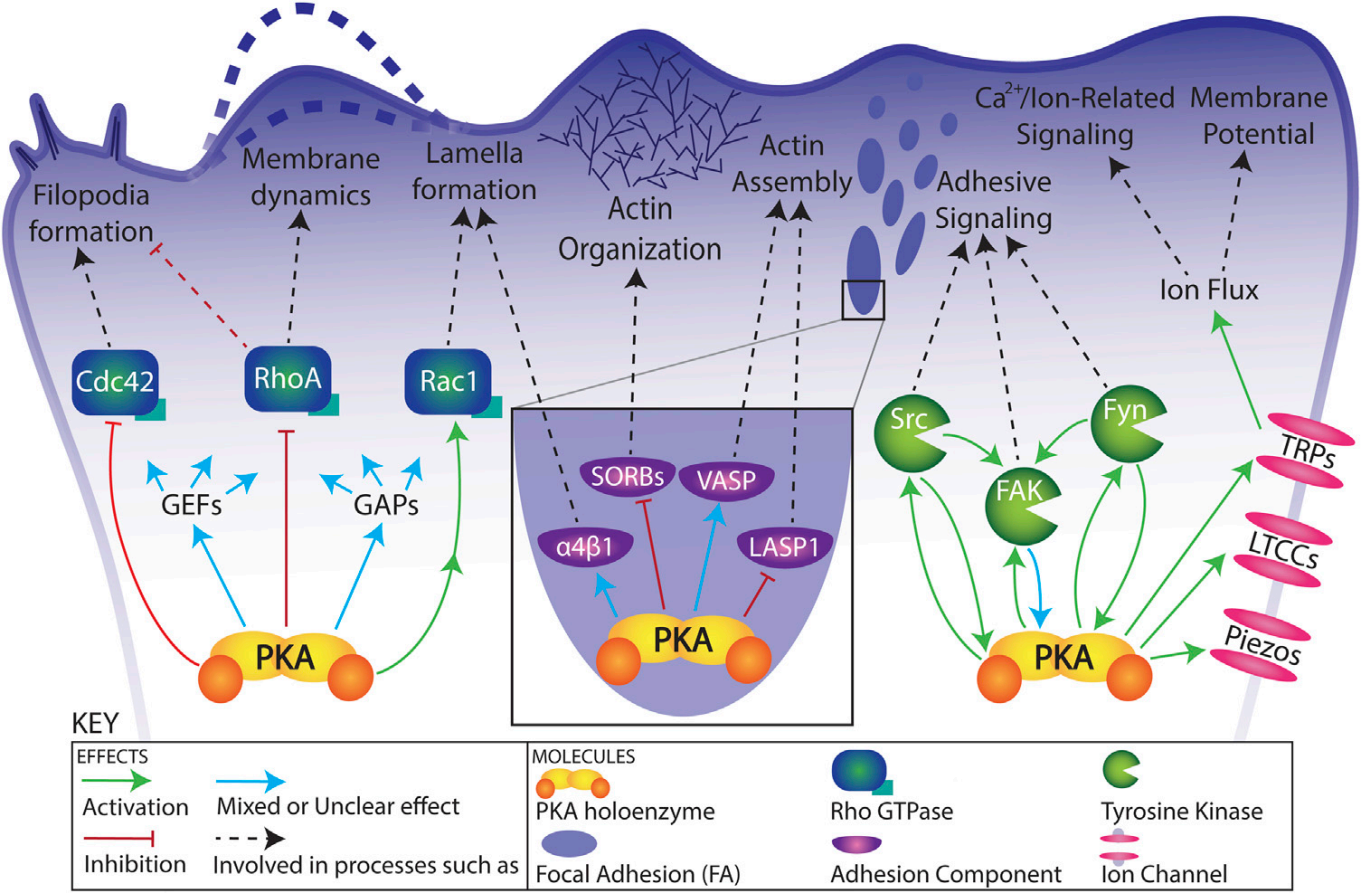
Schematic of functional connections between PKA and its targets in a migrating cell. Relationships are simplified for visual clarity. See text and Table 1 for details regarding functional connections.

Several Rho family GTPases are critical for the progression of cell migration (Lawson and Ridley, 2018). For examples of the seemingly paradoxical role of PKA activity on migration and the need for local fluctuations in PKA activity, one need not look further than Rho GTPases (Figure 2; Table 1). The following examples are all active in the leading edge during cell migration (Machacek et al., 2009), are critical to polarity, protrusion, lamella and filopodia formation, and cell migration (Lawson and Ridley, 2018), and are regulated by PKA activity.

**TABLE 1:** Migration-related targets of PKA activity.

Class	Substrate	Sites	Regulatory effect	Functional details	References
Rho GTPases	RhoA	S188	Inhibition	Promotes binding to RhoGDI and sequestration, regulates membrane protrusion/retraction cycles	Lang et al. (1996), Forget et al. (2002), (Tkachenko et al., 2011)
	Cdc42	S185^a^	Inhibition	Promotes binding to RhoGDI and sequestration	Forget et al. (2002)
	Rac1	-	Indirect activation	Activation in pseudopods and other contexts	O'Connor and Mercurio, (2001), Howe et al. (2005)
Rho GEFs and GAPs	ARHGAP17	S702	Activation, Binding partners	Decrease Rac1 activity, dissociation from a complex with Cdc42 effector CIP4, dynamic stimulation of cell migration	Nagy et al. (2015)
	*Rac1 specific GAP*				
	ARHGEF6	S684	Binding partners	Promotes binding of 14-3-3 to ARGHEF in complex with GIT1	Nagy et al. (2015)
	*Rac1 specific GAP*	*S640*			
	STEF/Tiam2	T749	Activation	Activation of Rac1, neurite outgrowth	Goto et al. (2011)
	*Rac1 specific GEF*	S782			
		S156			
	DOCK180	S1250	Activation	Activation of Rac1, promotion of cell migration	Feng et al. (2014), Feng et al. (2015)
	*Rac1 specific GEF*				
	P-Rex1	S436	Inhibition, Activation by PKA RI	Decreased Rac1 activity driven by phosphorylation via PKA catalytic subunit, increased Rac1 activity driven by PKA RI	Chávez-Vargas et al. (2016), Adame-García et al. (2019)
	*Rac1 specific GEF*				
	β_1_Pix	S516	Activation, Localization	Activation of Cdc42, translocation of β_1_Pix to FAs	Chahdi et al. (2005), Chahdi and Sorokin, (2006)
	*Cdc42 specific GEF*	T526			
	GEF-H1	S886	Binding partners, Inhibition	Inhibition of RhoA activity through increased binding to microtubules, increased association with 14-3-3	Comer et al. (2020)
	*Rho specific GEF*				
	Myo9b	S1354	Activation	Inhibition of RhoA activity	Comer et al. (2020)
	*Rho specific GAP*				
	AKAP-Lbc	S1565	Binding partners, Inhibition	Inhibition of RhoA activity through 14-3-3 binding	Diviani et al. (2004), Diviani et al. (2006)
	*Rho specific GEF*				
Focal Adhesion Components	VASP	S153	Mixed	Decreased control of actin dynamics, inhibited maturation of FAs, accretion at peripheral cellular structures	Howe, (2004), Benz et al. (2009), Lee and Chung, (2009)
		S235			
		T274			
	LASP1	S99	Binding partners,	Decreased affinity for F-actin, displacement from FAs, translocation to the nucleus	Chew et al. (1998), Chew et al. (2002), Butt et al. (2003), Keicher et al. (2004), Grunewald and Butt, (2008), Mihlan et al. (2013)
		S146	Localization		
	α4β1 integrins	S988 (α4)	Binding partners	Stabilization of lamellipodia at the leading edge, disruption of paxillin binding to α4 tail	Goldfinger et al. (2003)
	ArgBP2/SORBS2	S259	Binding partners	Phosphorylation causes 14-3-3 binding, disrupting binding with α-actinin and therefore ArgBP2 function at stress fibers, promoting cell migration	Anekal et al. (2015)
	vinexin/SORBS3	-	-	Involved in PKA-mediated anchorage-dependent signaling	Suwa et al. (2002)
Non-receptor Tyrosine Kinases	Src	S17	Increased catalytic activity	Conformational change resulting in exposure and phosphorylation of Y419 activating site, promotes ovarian cancer cell migration	Schmitt and Stork, (2002), Armaiz-Pena et al. (2013), Beristain et al. (2015)
	Fyn	S21	Increased catalytic activity, Localization	Increased activity and localization to FAs, promoting migration, FA dynamics, and leading edge dynamics	Yeo et al. (2011)
	FAK	-	Mixed	Indirect positive regulation through Src and Fyn, negative regulation in anchorage-dependent signaling, likely required for full FAK activation and cell migration	Howe and Juliano, (2000), Sanchez-Collado et al. (2019)
Ion Channels	L-type Calcium Channel Ca_v_1.2	S1928^b^	Increased channel activity, Binding Partners	Positive regulation of channel activity dependent on binding/scaffolding of several AKAPs including AKAP79 and AKAP Cypher/Zasp, changes binding of calmodulin, mediates calcium response to adrenoreceptor activation	Gray et al. (1998), Murphy et al. (2014), Nystoriak et al. (2017), Yu et al. (2018), Pallien and Klussmann, (2020)
		S1700^b^			
		T1704^b^			
		S1458			
	TRPV1	S116	Receptor sensitization	Phosphorylation dependent on scaffolding of TRPV1 with AKAP150	Rathee et al. (2002), Por et al. (2013), Mohapatra and Nau, (2003), Mohapatra and Nau, (2005)
		T144			
		T370			
	TRPV4	S824	Receptor sensitization	Phosphorylation dependent on scaffolding of TRPV4 with AKAP79	Fan et al. (2009)
	TRPM7	S1269^a^	Mixed	Phosphorylation at S1269 decreases Ca^2+^ influx, unidentified regulation downstream of PKA increases TRPM7 activity	Broertjes et al. (2019), Takezawa et al. (2004)
	TRPC6	T69	Inhibition	Decreased channel activity	(Nishioka et al., 2011), Horinouchi et al. (2012)
		S28			
	Piezo 2	-	Activation	Increased PKA activity increases Piezo 2 activity	Dubin et al. (2012)

a Insufficient evidence of direct phosphorylation.

b Residue numbering based off rabbit sequence.

#### 3.1.1 RhoA

RhoA is classically associated with contractility and the formation of actin stress fibers and focal adhesions (Ridley and Hall, 1992). Rho is a direct substrate of PKA phosphorylation (Lang et al., 1996). Historically, this phosphorylation was considered to inhibit binding of RhoA to Rho kinase, inhibiting Rho kinase (Dong et al., 1998). PKA phosphorylation of RhoA at Ser188 leads to inhibition of Rho membrane association (Lang et al., 1996). This dissociation from the membrane is achieved through increased association of RhoA with RhoGDI (Forget et al., 2002). RhoA is active at the foremost edge of the leading edge and its activity is correlated with membrane protrusion (Machacek et al., 2009). It is now generally understood that fluctuations in PKA activity control RhoA activity at the leading edge to promote extension of the cell membrane and protrusion/retraction cycles. In a study mentioned previously, using biosensors for both PKA and RhoA and protein biochemistry, PKA was found to regulate membrane protrusion-retraction cycles at the leading edge through its direct phosphorylation of RhoA and subsequent association of phosphorylated RhoA with RhoGDI (Tkachenko et al., 2011).

#### 3.1.2 Cdc42

Also active in the leading edge, Cdc42 is well known for its role in the extension of protrusions such as filopodia and microspikes (Machacek et al., 2009; Lawson and Ridley, 2018), structures known to concentrate PKA activity, as described previously. Like RhoA, Cdc42 is a direct substrate of PKA, but the functional consequences of this phosphorylation are not well explored. Multiple studies reveal that like that of RhoA, PKA mediated phosphorylation of Cdc42 increases its inactivation by association with RhoGDI (Forget et al., 2002; MacKeil et al., 2019), but the effects of PKA phosphorylation of Cdc42 remain overall less well characterized than that of RhoA.

#### 3.1.3 Rac1

Rac1 GTPase is critical for the formation of lamellipodia through regulation of actin polymerization and turnover (Lawson and Ridley, 2018). Rac1 possesses AKAP properties and active Rac forms a complex with and stabilizes the PKA holoenzyme (Bachmann et al., 2013a; Bachmann et al., 2013b). Rac1 is not generally regarded as a direct substrate of PKA [save for an observation in (Brandt et al., 2009)] and it lacks the serine residue involved in PKA phosphorylation and consequent RhoGDI sequestration of RhoA and Cdc42 (Forget et al., 2002). However, PKA activity is linked to activation of Rac1 (O'Connor and Mercurio, 2001; Dormond et al., 2002; Howe et al., 2005), demonstrating a functional connection between PKA and Rac1 in migration. This relationship has been given mechanistic foundations through the identification of several Rac GAPs [ARHGAP17 (activating/binding partners) (Nagy et al., 2015)] and Rac GEFs [ARHGEF6 (binding partners) (Nagy et al., 2015), STEF/Tiam2 (activating) (Goto et al., 2011), DOCK180 (activating) (Feng et al., 2014; Feng et al., 2015), and P-Rex1 (inactivating) (Chávez-Vargas et al., 2016)] that are directly phosphorylated by PKA. P-Rex1 is a particularly interesting target of PKA phosphorylation. In addition to regulation by direct phosphorylation by PKA, this Rac GEF is also a non-canonical AKAP which reciprocally regulates PKA by bringing it to the plasma membrane (Chávez-Vargas et al., 2016). Expression of a phospho-resistant mutant of P-Rex1 not only increased its activity but abrogated the migration-stimulating effect of PKA activation on endothelial cell migration (Chávez-Vargas et al., 2016). Interestingly, the inhibition of P-Rex1 by PKA catalytic subunit is complemented by an activation of P-Rex1 by PKA type I regulatory subunit (Adame-García et al., 2019). Given this interesting dichotomous regulation, one could argue that spatial regulation by PKA of P-Rex1 may be furthered by altered ratios of catalytic to regulatory subunits in the leading edge versus cell body as described in (Howe et al., 2005).

#### 3.1.4 Rho GEFs and GAPs

Finally, the regulation of other Rho GEFs and GAPs by PKA further implicates PKA as a master regulator of the activity of these molecular switches in migration (Figure 2; Table 1). This complexity is exemplified by PKA’s effects on Cdc42-specific GEF β_1_Pix (activation, localization) (Chahdi et al., 2005; Chahdi and Sorokin, 2006), Rho-specific GEF GEF-H1 (inactivation) and Rho-specific GAP Myo9b (activation) (Comer et al., 2020). Perhaps increasing its importance and relevance in the leading edge, AKAP-Lbc (described above in The leading edge) is a Rho-specific GEF and target of PKA phosphorylation (inactivating) (Diviani et al., 2004; Diviani et al., 2006). This type of multipurpose scaffolding molecule/effector is an excellent example of local contextual regulation of PKA and its targets.

### 3.2 Focal adhesion components

Many focal adhesion components have been implicated or confirmed to be targets of PKA phosphorylation. Given the localization of PKA to adhesive complexes, this abbreviated list of targets draws attention to the need for further investigation into the effects of PKA phosphorylation on adhesion dynamics (Figure 2; Table 1).

#### 3.2.1 VASP

Vasodilator-stimulated phosphoprotein (VASP), thoroughly reviewed in migration with its Ena/VASP family members in (Faix and Rottner, 2022), is a quintessential PKA substrate involved in adhesion and migration (Krause et al., 2003). Briefly, VASP and related proteins are involved in cytoskeletal dynamics as actin assembly factors and anti-capping proteins (Benz et al., 2009). VASP is essentially ubiquitous, like PKA, and it exists and exerts differing functions in different parts of the cell such as focal adhesions, the edge of lamellipodia, or tips of filopodia (Faix and Rottner, 2022). It's long been shown to be a direct substrate of PKA and the effects of this phosphorylation are not unilaterally inhibitory or stimulatory, rather VASP function and localization are modulated by phosphorylation by PKA (Howe, 2004). VASP phosphorylation by PKA is responsible for accretion of VASP at the cell periphery, in lamellipodia and focal adhesions, where dynamic actin remodeling is taking place (Benz et al., 2009) and this phosphorylation is dependent on PKA anchoring via ERM proteins (Deming et al., 2015). Unsurprisingly, VASP phosphorylation by PKA must be dynamic. Prolonged phosphorylation of VASP blocks maturation of focal adhesions (Lee and Chung, 2009). Thus, regulation of VASP by PKA can have different consequences and outcomes depending on precisely where and to what degree VASP is phosphorylated.

#### 3.2.2 LASP1

LASP1 is an F-actin-binding protein that localizes to FAs, lamellipodial edges, podosomes, and other microfilament-associated structures. It also translocates into the nucleus to regulate transcription (Butt and Raman, 2018). LASP1 has well-established and increasingly important roles in cell motility, cancer metastasis and prognosis (Ruggieri et al., 2017), neural development (Butt et al., 2003), and many other cellular functions (Grunewald and Butt, 2008; Butt and Raman, 2018). It is directly phosphorylated by PKA *in vitro* and *in vivo* (Chew et al., 1998; Chew et al., 2002; Butt et al., 2003; Keicher et al., 2004; Grunewald and Butt, 2008; Mihlan et al., 2013), and this modification decreases its affinity for F-actin (Chew et al., 2002; Butt et al., 2003), displaces it from FAs (Keicher et al., 2004), and facilitates its shuttling from the cytoplasm to the nucleus (Mihlan et al., 2013).

#### 3.2.3 α4β1 integrins

In addition to their AKAP function, α4β1 integrins are phosphorylated by PKA specifically in the leading edge of migrating cells (Goldfinger et al., 2003). Phosphorylation by PKA blocks paxillin binding to the tail of the α4 integrin. Further, this study showed that increased association of paxillin to α4, as occurs in the inhibition of PKA or elsewhere in the cell periphery, leads to destabilization of lamellipodia, stymying migration progress (Goldfinger et al., 2003). This spatially regulated phosphorylation of α4 by PKA is further required for the alignment of endothelial cells to shear stress and localized activation of Rac1 (Goldfinger et al., 2008).

#### 3.2.4 SORBS

Members of the SORBS adaptor protein family, specifically ArgBP2/SORBS2 and vinexin/SORBS3 (Kioka et al., 2002; Roignot and Soubeyran, 2009), are found in FAs and F-actin junctions, play important roles in motility, force generation and mechanotransduction (Kioka et al., 2002; Cestra et al., 2005; Ichikawa et al., 2017; Kuroda et al., 2018), and intersect with PKA as direct substrates and/or modulators of PKA-mediated anchorage-dependent signaling (Suwa et al., 2002; Anekal et al., 2015).

Given the number and variety of proteins found in focal adhesions (Zaidel-Bar et al., 2007; Kuo et al., 2011; Schiller et al., 2011; Horton et al., 2015) and that many of these components are known or putative PKA substrates (Robertson et al., 2015), it is likely that PKA may have myriad and complex roles in FA dynamics. Current efforts in our lab and others aim to expand our understanding of PKA’s roles in FA structures.

### 3.3 Non-receptor tyrosine kinases

Non receptor tyrosine kinases such as Src and Fyn, Src family kinases, and Focal Adhesion Kinase (FAK) are critical to integrin mediated adhesion and cell migration (Klinghoffer et al., 1999; Cary et al., 2002; Mitra et al., 2005; Yeo et al., 2011). Though classically thought of as distinct from one another, connections and crosstalk between the cAMP/PKA pathway and tyrosine kinase pathways have been identified more and more over the past decade, most commonly with PKA acting upstream of tyrosine kinase activity, but increasingly the other way around (Figure 2; Table 1).

#### 3.3.1 Src

Direct serine phosphorylation of Src increases Src activity and downstream tyrosine phosphorylation (Schmitt and Stork, 2002; Beristain et al., 2015). Serine phosphorylation at this site, downstream of PKA, promotes ovarian cancer cell migration (Armaiz-Pena et al., 2013). Further, Src activity and subsequent activation of FAK can be inhibited by PKA acting through C-terminal Src kinase (Csk) in membrane microdomains where all of the relevant signaling molecules coalesce (Abrahamsen et al., 2003). Similar effects are realized through Csk downstream in T cell activation (Vang et al., 2001) and vascular sprouting (Jin et al., 2010). Importantly, as has been shown for the EGF receptor (Caldwell et al., 2012), Src family kinases can phosphorylate PKA and this modification increases its catalytic activity (Schmoker et al., 2018).

#### 3.3.2 Fyn

Phosphorylation of Fyn by PKA alters its tyrosine kinase activity, localization to focal adhesion structures, and facilitates cell migration (Yeo et al., 2011). Disruption of this phosphorylation led to decreased migration and defective leading edge dynamics. Further, this phosphorylation of Fyn is critical for FAK activation and targeting to focal adhesions (Yeo et al., 2011).

In the reverse direction, tyrosine phosphorylation of PKA by Fyn increases PKA activity and changes PKA complexing with binding partners such as AKAPs and phosphodiesterases, which further complex with Fyn in a glioblastoma cell line (Schmoker et al., 2018).

#### 3.3.3 FAK

In addition to the indirect effects of PKA activity on FAK through Src and Fyn, as mentioned above, PKA negatively regulates FAK tyrosine phosphorylation in anchorage-independent signaling (Howe and Juliano, 2000). Adenylyl cyclase 8, presumably upstream of PKA activity, is required for full FAK activation and cell migration in MDA-MB-231 cells (Sanchez-Collado et al., 2019). Despite these observations, there are currently no published data supporting the converse relationship, placing FAK upstream of PKA activity. However, this is likely an important avenue of investigation given the roles of FAK and PKA in migration, mechanosensation, and cancer progression (Mitra et al., 2005; McKenzie et al., 2011; Sulzmaier et al., 2014; Hytonen and Wehrle-Haller, 2016; McKenzie et al., 2020).

### 3.4 Ion channels

Lastly, there are many known connections between PKA and several classes of ion channels (Gray et al., 1998; Fraser and Scott, 1999; Howe, 2011; Soni et al., 2014; Omar and Scott, 2020; Pallien and Klussmann, 2020). Particularly intriguing among these are L-type Ca^2+^ channels, TRP-family channels, and Piezo channels (Figure 2; Table 1).

#### 3.4.1 L-type Ca^2+^ channels

L-type Ca^2+^ channels (LTCCs) are responsible for retraction at the trailing edge (Yang and Huang, 2005), a front to rear Ca^2+^ gradient that maintains cell polarity (Kim et al., 2016), regulation of filopodia stability (Jacquemet et al., 2016), mechanosensation in filopodia (Efremov et al., 2022), and other functions relating to cell migration (Cheli et al., 2016; Martínez-Delgado and Felix, 2017; Guo et al., 2018; Kamijo et al., 2018; Birey et al., 2022). LTCC activity is positively regulated by PKA which is anchored to signaling scaffolds surrounding LTCCs via AKAP79, AKAP Cypher/Zasp, and others (Gray et al., 1998; Flynn and Altier, 2013; Murphy et al., 2014; Nystoriak et al., 2017; Smith et al., 2018; Yu et al., 2018; Pallien and Klussmann, 2020). As LTCCs have been shown to be critical for the sensory function of filopodia (Jacquemet et al., 2016; Efremov et al., 2022), a structure in which PKA has been shown to localize and function (Nauert et al., 1997; Han et al., 2007; Burgers et al., 2012), a hypothesis arises that a PKA-AKAP79-LTCC complex may be found within these structures.

#### 3.4.2 Transient receptor potential channels

Transient Receptor Potential (TRP) channels, particularly TRPC6 (Weber et al., 2015; Farmer et al., 2019; Asghar and Törnquist, 2020), TRPV1 (Miyake et al., 2015), TRPV4 (Mrkonjić et al., 2015; Li et al., 2020; Yang et al., 2020; Lakk and Križaj, 2021), and TRPM7 (Clark et al., 2006; Su et al., 2006; Wei et al., 2009; Wang et al., 2014; Broertjes et al., 2019; Lefebvre et al., 2020; Yankaskas et al., 2021) are increasingly recognized as important regulators of cellular migration, as thoughtfully reviewed in (Howe, 2011; Fiorio Pla and Gkika, 2013; Canales et al., 2019). Importantly, all of the aforementioned channels have been shown to be either direct substrates of PKA [TRPV1 (Rathee et al., 2002; Mohapatra and Nau, 2003; Mohapatra and Nau, 2005; Por et al., 2013), TRPV4 (Fan et al., 2009; Cao et al., 2018), TRPC6 (Nishioka et al., 2011; Horinouchi et al., 2012), and likely TRPM7 (Tian et al., 2018; Broertjes et al., 2019)] or regulated downstream of PKA activity (TRPM7 (Takezawa et al., 2004), establishing these and possibly other members of the TRP channel family as important players in PKA-mediated ion flux during migration. Crosstalk between PKA and TRP channels during cell migration has been well documented and is reviewed in (Howe, 2011).

#### 3.4.3 Piezo channels

Piezo channels are massive, mechanically sensitive ion channels known to transmit mechanical signals, activate integrins, and regulate cell migration in several ways (Gottlieb, 2017; Nourse and Pathak, 2017; Canales Coutino and Mayor, 2021; Dombroski et al., 2021; Holt et al., 2021). PKA activity is potentiated by calcium influx downstream of piezo1 in confined migration (Hung et al., 2016) and piezo2 activity is enhanced by increased PKA activity (Dubin et al., 2012), suggesting a link between PKA and piezo channels in migration.

Clearly, given the number and variety of targets within these various cellular contexts, it is a vast oversimplification to think of PKA as either a positive or negative regulator of cell migration. PKA needs to be tightly and locally regulated to act on the correct targets to facilitate cell migration. This idea meshes well with the very nature of cell migration. Cell migration itself is a process of balance and of give and take. Cells must protrude and lay down new adhesive structures in some places and contract and disassemble contacts in others. At first glance, it may appear that PKA’s role in migration is messy, but, in fact, there is a simplicity and elegance to way a cell can express a single family of kinases that then acts throughout the cell according to context and local signals to carry out innumerable, specific local functions.

## 4 Regulation

Though PKA has been studied for decades and even its name, cAMP dependent protein kinase, implies its regulation has been sorted, not enough is known on the subcellular and micro regulation of its activity. In many cases it’s not evident which class of PKA is doing the work of signaling during migration, as the biosensors and assays do not generally distinguish between them. However, it is quite clear that both classes–type I and type II–of PKA activity can contribute to migration-specific signaling (Howe et al., 2005; Lim et al., 2007; McKenzie et al., 2011; Adame-García et al., 2019). Given that the major differences between types I and II PKA are the concentration of cAMP required for activation and the mostly (but not always) distinct anchoring proteins associated with them, type I vs. type II PKA signaling may hold as yet undetermined importance for subcellular regulation.

As discussed previously, the first layer of PKA regulation often occurs by binding of the catalytic subunit by the regulatory subunits, an interaction disrupted by the availability of cAMP. cAMP is produced by adenylyl cyclases (ACs), often downstream of G protein coupled receptor activation and G protein Gαs (Neer, 1995). ACs can also be directly inhibited by Gαi (Neer, 1995) and regulated both positively and negatively by Gβγ (Tang and Gilman, 1991; Taussig et al., 1994; Sunahara and Taussig, 2002; Diel et al., 2006), creating a complex combinatorial network of regulators of AC. In addition, there are also reports of cAMP-independent activation of PKA, adding an additional layer of complexity onto the matter (Dulin et al., 2001; Niu et al., 2001; Ferraris et al., 2002; Kopperud et al., 2003; Ma et al., 2005; Kohr et al., 2010). Thus, when one considers localized PKA activity in the context of cell migration, one must also consider localized control of the various upstream regulators of PKA in these contexts and niches, and the pathways that connect those regulators to the machinery of migration.

PKA holoenzymes are docked to specific locations within the cell through their interactions with AKAPs which can serve as higher order scaffolds that bring together many components of the cAMP pathway (Pidoux and Tasken, 2010; Stangherlin and Zaccolo, 2011). This allows for local regulation of PKA activity in other processes, but the specifics of the regulation of PKA in *migration* remain relatively unexplored. Generally, studies that identify a role for PKA in migration stop short of tackling the mode of spatial and temporal regulation, apart from identification of AKAP-based localization. Therefore, many questions remain as to how PKA is delivered to sites of activation in migration and how it is activated locally to achieve its specific functions therein.

The strong link between α4β1 integrins and PKA activity in the leading edge is certainly intriguing, but the details of PKA regulation in this context remain unknown. Using cAMP sensitive biosensors, leading edge gradients of cAMP have been observed (Lim et al., 2008). Though they do not categorically show that this cAMP gradient is driving PKA activity directly, parsimony would suggest that it is. Even if it is simply local cAMP that drives leading edge PKA, the mechanism by which cAMP production is spatially regulated is still unknown. Most attempts at directly inhibiting leading edge PKA use the rather heavy-handed application of PKA inhibitor H89. H89 is hardly specific to PKA (Davies et al., 2000) and acts directly at the level of the catalytic subunit, not interfering with cAMP availability or binding.

Engagement of β1 integrins and application of mechanical stress at the points of integrin engagement have been shown to activate G protein Gαs and lead to local increases in cAMP (Meyer et al., 2000; Alenghat et al., 2009). This gating of cAMP production by integrins and mechanical stress is certainly intriguing, particularly because leading edge PKA activity has been shown to be tightly coupled to mechanical inputs (McKenzie et al., 2020). Treatment with a potent inhibitor of myosin II (and thereby actomyosin contractility) diminishes leading edge PKA activity in under a minute. Further, PKA activity can be potentiated by application of mechanical stretch on a 2D hydrogel. Finally, PKA activity is required for durotaxis, or mechanically gated cell migration (McKenzie et al., 2020). The mechanism underlying the mechanical regulation of PKA is still under investigation. Cell migration itself is a process driven by iterative mechanical inputs as the cell constantly forms new connections and probes the extracellular environment for elasticity and structure (Plotnikov et al., 2012; Wong et al., 2014), so it stands to reason that the regulation of PKA is guided by this iterative mechanical probing.

Recalling the study using rapamycin-inducible recruitment of RII subunit, this manipulation had differing effects on PKA activity and directional cell migration depending on the level of induction (LaCroix et al., 2022). Moderate amounts of recruitment of R subunit to the membrane led to increased and sustained PKA activity there whereas high levels ultimately inhibited PKA activity (LaCroix et al., 2022). Though not completely unexpected, this result highlights the complexity of PKA regulation—location, relative abundance of subunits, and availability of upstream activators, binding partners, and targets coalesce to create higher order signaling complexes that bring PKA specifically to bear on a variety of processes involved in cell migration.

If we are to understand how PKA functions in the complex and contextual way outlined in this review, it is important to drill down and explore what regulators of adhesion and migration are proximally involved in regulating PKA activity. If the PKA activity in question is, indeed, regulated through the canonical pathway, there must be communication between adhesive complexes, cytoskeletal structures, and other migratory nodes and cyclases/phosphodiesterases. This regulation will be very tightly controlled and highly contextual. There is not a lot known about these connections, and for good reason. PKA regulation on this level is highly context specific in that it needs to be studied at very high conceptual and practical resolution. Needless to say, studying such a ubiquitous kinase at a granular, subcellular level is complex, but identifying signaling niches and the regulatory machinery within those niches will be key to understanding how PKA functions in this contextual manner during cell migration.

## 5 Discussion

Gradients and other heterogeneities in the extracellular environment must be converted into asymmetries in the intracellular biochemical processes that iteratively reshape and reposition the cell to achieve cell movement. Understanding this conversion requires detailed understanding of the interface between signaling enzymes and cytoskeletal and adhesive structures/machineries that execute the physical steps of cell migration. Though PKA signaling is widely recognized as important for migration and this topic has been the focus of a fair number of studies throughout the past two decades, the sum of these studies merely scratches the surface of the complexity therein. Anchored PKA complexes function as tethered, multi-component sensors/relays for converting regional extracellular stimuli into specific, precise, and highly localized intracellular effects. Many such complexes, as well as known and putative PKA substrates, are found in a variety of subcellular compartments and structures involved in cell migration. Thus, elucidation of the composition, regulation, and precise function of these distinct PKA signaling complexes is an important endeavor in understanding of the complexity of spatial regulation of migration.
